# A Novel Dual Allosteric Activation Mechanism of *Escherichia coli* ADP-Glucose Pyrophosphorylase: The Role of Pyruvate

**DOI:** 10.1371/journal.pone.0103888

**Published:** 2014-08-07

**Authors:** Matías D. Asención Diez, Mabel C. Aleanzi, Alberto A. Iglesias, Miguel A. Ballicora

**Affiliations:** 1 Department of Chemistry, Loyola University Chicago, Chicago, Illinois, United States of America; 2 Laboratorio de Enzimología Molecular, Instituto de Agrobiotecnología del Litoral (UNL-CONICET), FBCB Ciudad Universitaria, Santa Fe, Argentina; Centre National de la Recherche Scientifique, Aix-Marseille Université, France

## Abstract

Fructose-1,6-bisphosphate activates ADP-glucose pyrophosphorylase and the synthesis of glycogen in *Escherichia coli*. Here, we show that although pyruvate is a weak activator by itself, it synergically enhances the fructose-1,6-bisphosphate activation. They increase the enzyme affinity for each other, and the combination increases *V*_max_, substrate apparent affinity, and decreases AMP inhibition. Our results indicate that there are two distinct interacting allosteric sites for activation. Hence, pyruvate modulates *E. coli* glycogen metabolism by orchestrating a functional network of allosteric regulators. We postulate that this novel dual activator mechanism increases the evolvability of ADP-glucose pyrophosphorylase and its related metabolic control.

## Introduction

Both glycogen synthesis in bacteria and starch synthesis in plants share a key metabolic step: synthesis of the glucosyl-donor ADP-glucose (ADP-Glc). The reaction is catalyzed by ADP-Glc pyrophosphorylase (EC: 2.7.7.27; ADP-Glc PPase), which is allosterically regulated by metabolites from the main carbon assimilation route in the respective organism [1], [2]. It belongs to an enzyme family with kinetic properties adapted to different metabolic environments. This is evidenced by a certain degree of promiscuity observed for the substrate and/or activator in some of the groups [3], [4]. Pyr was previously reported as weak activator for the enzyme from enterobacteria [5]. However, almost no kinetic data regarding Pyr activation was collected and no physiological relevance was inferred. In this work we found an important role for Pyr in the *E. coli* ADP-Glc PPase.

ADP-Glc PPase catalyzes the reaction ATP+Glc-1P ⇌ ADP-Glc+PP_i_ in the presence of Mg^2+^ [1], [2]. The enzyme activators are small molecules that indicate high energy within the cell, whereas the inhibitors indicate starvation [1], [2], [6]. The whole regulatory scenario is compatible with an enzyme involved in synthesis of cellular reserves of carbon and energy, which uses ATP as a substrate.

The crystal structures of the enzyme from *A. tumefaciens* and the small subunit from potato tuber have been solved, but the regulatory mechanism remains largely unknown [7], [8]. Several studies have established structure-function-regulation relationships between ADP-Glc PPases from different organisms, and those studies showed enzymes with different specificity for different regulators [1], [2], [9], [10]. Despite the broad diversity in regulator specificity in different species and metabolic environments, we recently identified key common regulatory loops conserved throughout the ADP-Glc PPase family. They are involved in propagating the allosteric signal both in *E. coli* [11] and potato tuber [12]. This indicated that the same allosteric mechanism, but with different effectors, could be shared among very distant species from bacteria and plants.

The allosteric regulatory properties of the *E. coli* ADP-Glc PPase has been extensively characterized, where Fru-1,6-P_2_ and AMP are the main activator and inhibitor, respectively [1], [11], [13]–[19]. Early studies acknowledged that the *E. coli* enzyme has a series of other minor allosteric regulators [17], [20]. One of those is Pyr, which produces a weak activation of the enzyme with an *A*_0.5_ higher than 10 mM [5]. This value suggested that this keto acid was not of physiological relevance for glycogen synthesis regulation in enterobacteria [1], [2]. However, in the last decade, it has been found that Pyr is at relatively high levels in *E. coli* and it is critical to control metabolic fluxes [21]. For that reason, the role of the keto acid in enterobacteria as regulator of the polysaccharide metabolism required re-examination.

Herein, we report a detailed study on the kinetic effects exerted by Pyr on the *E. coli* ADP-Glc PPase. These results lead us to reconsider the relevance of the metabolite as a modulator of the enzyme activity and allow us to propose a novel dual allosteric mechanism. Pyr interacts with the established main effectors and reciprocally strengthens the allosteric control.

## Materials and Methods

### Chemicals, enzymes and bacterial strains

Protein standards, antibiotics, isopropyl-*β*-thiogalactoside (IPTG), substrates, and inorganic pyrophosphatase were from Sigma-Aldrich (Saint Louis, MO, USA). Stocks solutions of Pyr were prepared and taken to pH 8.0 before adding it to the reaction mixture to avoid pH changes at the highest concentrations. All the other reagents were of the highest quality available. The ADP-Glc PPase was expressed from pETEC (pET24a plasmid derivative) using *E. coli* BL21 (DE3) cells, and then purified to apparent electrophoretic homogeneity as described elsewhere [5], [22].

### Protein methods

Denaturing protein electrophoresis (SDS-PAGE) was conducted as described by Laemmli [23]. Protein concentration was determined by absorbance at 280 nm with a NanoDrop 1000 (Thermo Scientific, Wilmington, DE) using an extinction coefficient of 1.273 ml mg^−1^ cm^−1^, determined from the amino acid sequence by using the ProtParam server (http://web.expasy.org/protparam/) [24].

### Enzymatic Assays

Synthesis of ADP-Glc was assayed by following the formation of Pi (after hydrolysis of pyrophosphate by inorganic pyrophosphatase) as previously described [25]. Unless otherwise stated, a 50 µl reaction mixture contained 100 mM HEPES (pH 8.0), 10 mM MgCl_2_, 1.5 mM ATP, 0.2 mg/ml bovine serum albumin, 0.5 mU/µl yeast inorganic pyrophosphatase, and the given amount of enzyme. Assays were initiated with 1 mM Glc-1P. If mentioned, a given amount of Pyr, Fru-1,6-P_2_, and/or AMP was also included. After incubation for 10 min at 37°C, addition of Malachite Green reagent terminated the reaction. The complex formed with the released P_i_ was measured at 630 nm with an ELISA EMax detector (Molecular Devices). Sodium pyrophosphate was used as standard. One unit of enzyme activity was defined as the amount producing of 1 µmol of product in 1 min under the specified conditions.

### Kinetic characterization

Data of enzyme activity were plotted versus effector concentration. Kinetic parameters from the Hill equation such as Hill coefficient (*n*_H_), maximal velocity (*V*_max_), as well as the activator substrate or inhibitor concentrations giving 50% of the maximal activation (*A*_0.5_), velocity (*S*_0.5_), or inhibition (*I*_0.5_), were acquired by fitting the data with a non-linear least-squares algorithm using the program Origin 7.0 (OriginLab). Parameters are the mean of at least three independent sets of data, reproducible within ±10%. Sample standard deviations of the data were calculated from the Hill equation fitting by using the Levenberg–Marquardt method.

## Results

### Pyr activates *E. coli* ADP-Glc PPase

**Figure 1:**
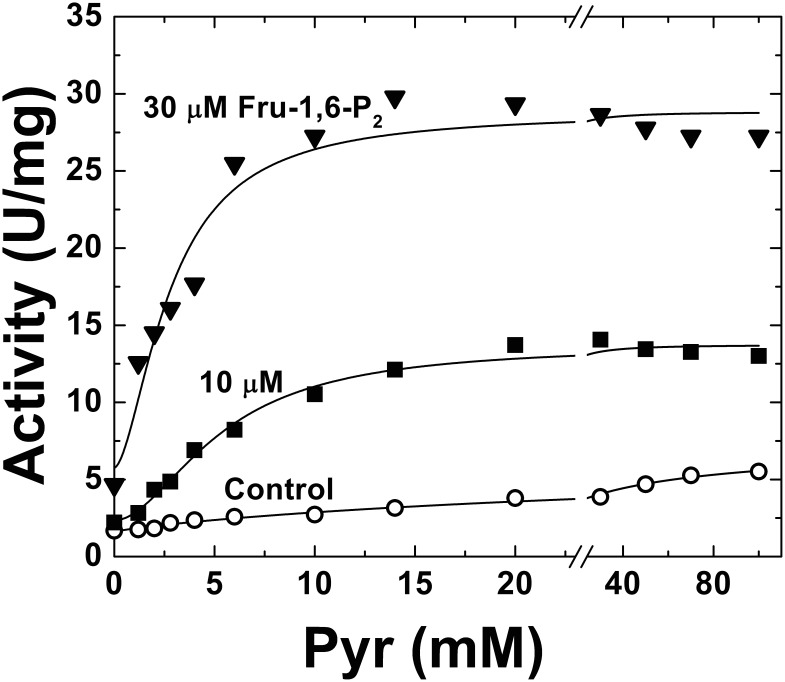
*E. coli* ADP-Glc PPase Pyr saturation curves in the absence (control, ○) or presence of Fru-1,6-P_2_ (10 µM, ▪; or 30 µM, ▾). Other conditions for the assays were as described under Materials and Methods. •□▴.

Pyr has been reported as a very weak activator of the *E. coli* ADP-Glc PPase [5], but its regulatory effect has not been thoroughly investigated. For that reason, we decided to first examine the effect of Pyr in the absence of any other regulatory effector. A detailed study of the enzyme activity (assayed in the physiological direction of ADP-Glc synthesis) in presence of increasing Pyr concentrations is depicted in Figure 1. Pyr increased the enzyme activity 3.3-fold at the highest concentrations tested (100 mM), following hyperbolic (n_H_ value of 1.03) kinetics with an *A*_0.5_ of 25 mM. In addition, Pyr increased the apparent affinity of the enzyme for the substrates (Table 1, assay condition A, B and C). The *S*_0.5_ for ATP was reduced 3.9- or 6.2-fold by the presence of Pyr at 25 mM or 50 mM, respectively. Similar, although slightly lower, was the effect of Pyr on the other substrate, Glc-1P. In that case, the *S*_0.5_ for Glc-1P was lowered 1.7- and 2.6-fold (Table 1).

**Table 1:** Kinetic parameters for *E. coli* ADP-Glc PPase.

Assay Condition	Glc-1P		ATP-Mg		*V*_max_ (U/mg)
	*S*_0.5_ (mM)	*n*_H_	*S*_0.5_ (mM)	*n*_H_	
A. No effector	0.95±0.07	1.1	6.49±0.21	1.5	1.67±0.15
B. 25 mM Pyr	0.57±0.05	1.0	1.67±0.08	2.5	3.93±0.21
C. 50 mM Pyr	0.37±0.02	0.9	1.05±0.06	2.2	5.31±0.19
D. 25 mM Pyr +10 µM Fru-1,6-P_2_	0.23±0.02	1.3	0.99±0.07	3.6	31±2
E. 10 µM Fru-1,6-P_2_	0.77±0.04	1.0	5.02±0.25	2.1	6.21±0.19
F. 50 mM Pyr +150 µM Fru-1,6-P_2_	0.09±0.01	1.1	0.37±0.03	2.4	104±5
G. 150 µM Fru-1,6-P_2_	0.12±0.01	1.2	1.31±0.09	3.2	63±4
I. 2 mM Fru-1,6-P_2_	0.09±0.01	1.3	0.43±0.02	2.1	108±6
J. 50 mM Pyr +2 mM Fru-1,6-P_2_	0.11±0.01	1.0	0.19±0.02	1.4	110±5

Assays were carried out as described in Material and Methods. Values are average numbers from three independent experiments, using regression analysis.

As a whole, the effect of Pyr on the *E. coli* ADP-Glc PPase is not significant if compared to Fru-1,6-P_2_, which increases the enzyme activity by near ∼50-fold with an *A*_0.5_ in the sub-millimolar range [11], [14], [17]. The apparent affinity for Pyr seemed to be poor. Nonetheless, this keto acid not only increased the enzyme maximal activity, but also the affinity for substrates. In fact, the ∼4-fold increase in *V*_max_ of the *E. coli* ADP-Glc PPase resembles the 5-fold Pyr activation of the *A. tumefaciens* enzyme. The main difference is that the *A. tumefaciens* enzyme has an apparent affinity for the keto acid more than two orders of magnitude higher (*A*_0.5_ of 0.13 mM) and that Fru-6P is the other major activator with comparable kinetic parameters [26].

### Interplay between Pyr and Fru-1,6-P_2_

**Figure 2:**
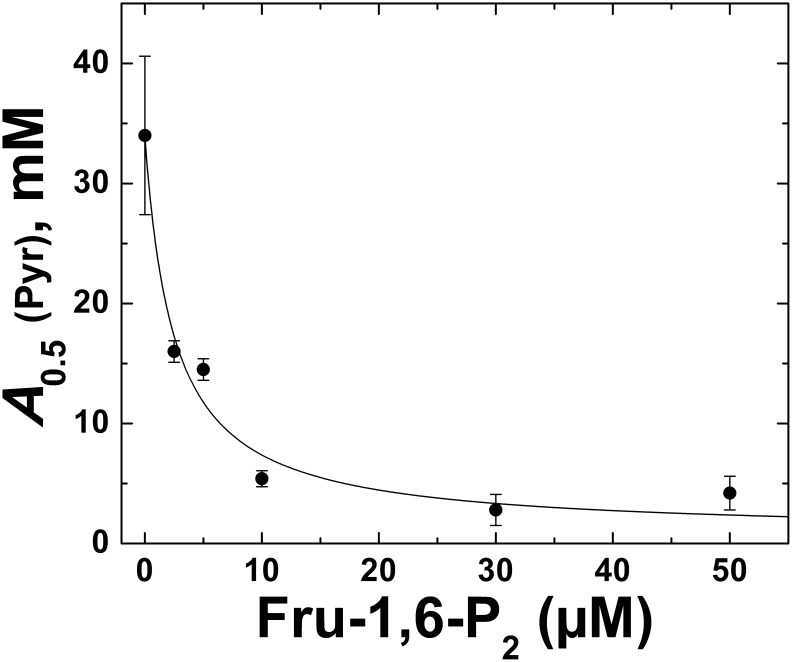
Effect of Fru-1,6-P_2_ on the apparent affinity of ADP-Glc PPase for Pyr. Other conditions for the assays were as described under Materials and Methods. Values are the mean of three independent measurements ± standard deviation.

To determine whether Pyr has a synergistic effect or another type of interaction with other regulators we tested it in presence of the main allosteric effector of the enzyme (Fru-1,6-P_2_) at concentrations below the *A*_0.5_. As shown in Figure 1, the effect of Pyr as allosteric activator of the *E. coli* ADP-Glc PPase was enhanced when analyzed in presence Fru-1,6-P_2_. Even at sub-saturating concentrations of 10 µM and 30 µM, Fru-1,6-P_2_ improved the apparent affinity of the enzyme for Pyr 4.9- and 8.4-fold, respectively. In those conditions, the keto acid produced a maximal activation of ∼6-fold (Figure 1). These results prompted us to further analyze the combination of both activators and its effect on the enzyme behavior. The increment in the *E. coli* ADP-Glc PPase apparent affinity for Pyr was clearly observed at increasing concentrations of Fru-1,6-P_2_ (Figure 2). The *A*_0.5_ for Pyr went down from 34 mM to a low mM range. Remarkably, a Fru-1,6-P_2_ concentration as low as 10 µM (∼10 times lower than the *A*_0.5_) more than doubled the Pyr apparent affinity. This clearly indicates a synergistic effect between Pyr and Fru-1,6-P_2_.

To establish whether this synergy affected the interaction with the substrates, we determined the ATP and Glc-1P apparent affinities in presence of Pyr (25 or 50 mM) and sub-saturating, slightly above half-saturation, or saturating concentrations of Fru-1,6-P_2_ (10, 150 or 2000 µM, respectively). As illustrated in Table 1 (D), the combination of activators enhanced the affinity toward the enzyme substrates. This effect was higher than the ones produced by each of them separately (Table 1, C & E). Thus, 10 µM Fru-1,6-P_2_ alone only slightly (1.2- to 1.3-fold) modified the affinity for either of the substrates. However, when combined with 25 mM Pyr, the affinity for Glc-1P increased 4.1-fold (compared to values in absence of any effector). On the other hand, the increase caused by 25 mM Pyr alone was only 1.7-fold. As well, the presence of both activators lowered the *S*_0.5_ for ATP 6.6-fold, whereas 25 mM Pyr in absence of Fru-1,6-P_2_ decreased it 3.9-fold. In addition, saturating concentrations of both activators (Table 1, assay condition F) produced the highest apparent affinities for substrates in the *E. coli* ADP-Glc PPase, indicating that Pyr enhanced the activation exerted by Fru-1,6-P_2_ alone (Table 1, condition G).

**Figure 3:**
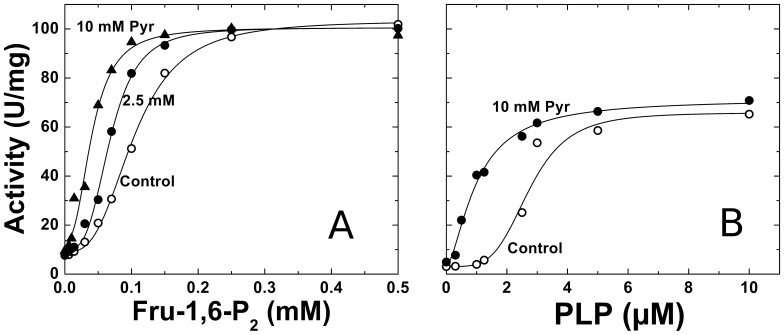
A) Effect of Pyr on the saturation curves for Fru-1,6-P_2_. Assays were performed in the absence (control, ○), or the presence of Pyr (2.5 mM, •; or 10 mM, ▴). B) Effect of Pyr on the saturation curves for PLP. Assays were performed in the absence (control, ○), or the presence of 10 mM Pyr (▴). Other conditions for the assays were as described under Materials and Methods.

The Pyr to Fru-1,6-P_2_ synergistic interaction is also reinforced by results showed in Figure 3. We analyzed the *E. coli* ADP-Glc PPase response to Fru-1,6-P_2_ when the enzyme was in absence or presence of sub-saturating concentrations of Pyr (Figure 3A). The concentration of Pyr, 2.5 mM, was 10-fold lower than its *A*_0.5_. Notably, this Pyr concentration, which has no prominent intrinsic effect on the *E. coli* ADP-Glc PPase, doubled the Fru-1,6-P_2_ apparent affinity (the Fru-1,6-P_2_ *A*_0.5_ value changed from 0.12 mM to 0.07 mM). In addition, Pyr clearly increased the enzyme sensitivity to pyridoxal-5′-phosphate (PLP) (Figure 3B). In previous works, it was demonstrated that PLP is incorporated to the *E. coli* ADP-Glc PPase emulating Fru-1,6-P_2_ activation by binding to Lys^39^ [27]–[29]. In that respect, PLP has been extensively used as an analog of activators, not only in enzymes activated by Fru-1,6-P_2_, but also by 3-phosphoglycerate. Indeed, the enzyme became more susceptible to PLP in presence of 10 mM Pyr; not only with a decrease in the *A*_0.5_ value (from 2.7 µM to 1.0 µM), but also with a dramatic change (from sigmoidal to hyperbolic) in the activation pattern (Figure 3B). In addition, we evaluated the Pyr activation of the *E. coli* ADP-Glc PPase in presence of 1 µM PLP, which has no direct effect on the enzyme. In this condition, the *A*_0.5_ for the keto acid was as low as 3.7 mM (data not shown). This indicated that a sub functional concentration of PLP lowered the *A*_0.5_ for Pyr one order of magnitude. This result agrees with the observed increase in the affinity for Pyr caused by Fru-1,6-P_2_ (Figure 1 and Table 1). As a whole, it could be inferred that Pyr at lower concentrations makes the enzyme more sensitive to Fru-1,6-P_2_, and *vice versa*.

### Pyr diminishes the inhibition by AMP

**Figure 4:**
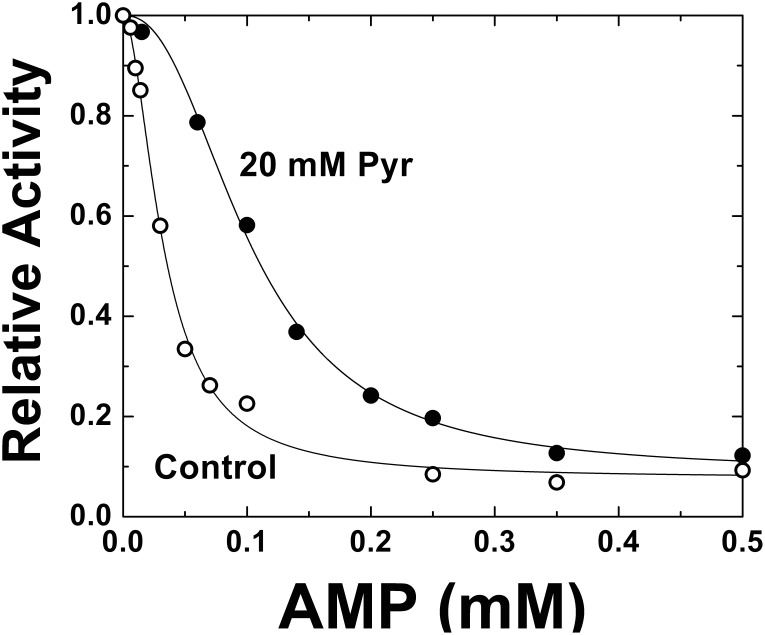
Effect of Pyr on the AMP inhibition. Assays were performed in the absence (control, ○), or the presence of 20 mM Pyr (•). Other conditions for the assays were as described under Materials and Methods. Activity is relative to experiments conducted in absence or presence of Pyr, whose absolute values were 31.2 and 61.8 U/mg, respectively.

It is well known that AMP is the major inhibitor of the *E. coli* ADP-Glc PPase, mainly acting in a cross-talk with the activator Fru-1,6-P_2_ [1], [14], [18], [19]. To advance in the characterization of the role of Pyr, we analyzed how this metabolite affected the kinetic behavior of AMP inhibition of the *E. coli* ADP-Glc PPase. Curves of AMP were obtained in the absence or in the presence of 20 mM Pyr. In all cases reaction mixtures contained 100 µM Fru-1,6-P_2_ because the activator is needed to observe AMP inhibition [20]. It is important to note that this value is near the *A*_0.5_ for Fru-1,6-P_2_ (Figure 3), which is high enough to have a significant activation, but low enough to allow a reversion by AMP. Results depicted in Figure 4 clearly indicate that when Pyr was present the enzyme was less sensitive to AMP inhibition. Pyr diminished the apparent affinity for AMP 3-fold. The *I*_0.5_ for AMP changed from 0.03 mM, a typical value for this condition [1], [14], to 0.11 mM. Also, the maximum inhibition was very similar in both cases (around 10% of remaining activity). However, in presence of Pyr, the enzyme depicted a more sigmoidal behavior, where *n*_H_ changed from 1.7 to 2.4. When the relative activities from both inhibition curves were compared it was observed that *E. coli* ADP-Glc PPase was up to 3-fold more active in the 0.05–0.15 mM AMP range if Pyr was present.

**Figure 5:**
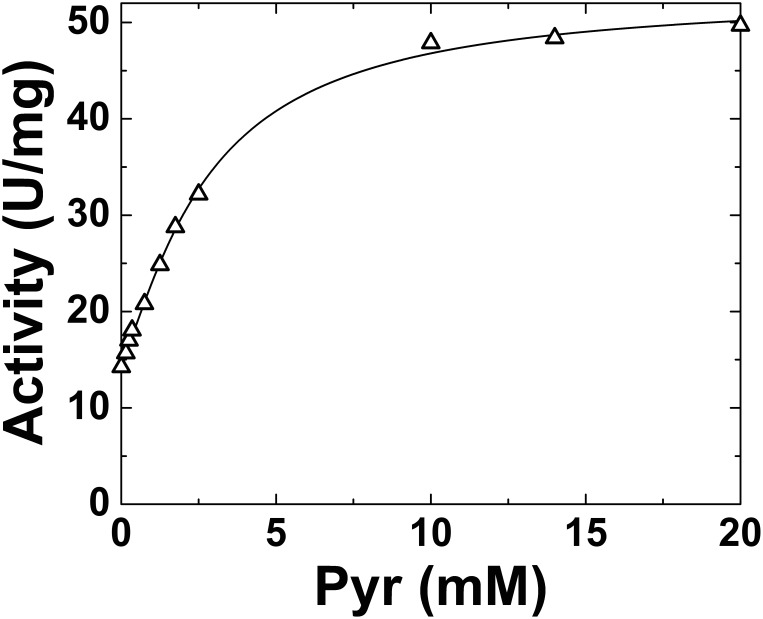
Partially inhibited *E. coli* ADP-Glc PPase is activated by Pyr. The enzyme activity was determined in presence of 100 µM Fru-1,6-P_2_ and 50 µM AMP. These concentrations were the *A*_0.5_ and the *I*_0.5_ values, respectively, as this range maximized the sensitivity of the enzyme for these effectors. Other conditions for the assays were as described under Materials and Methods.

We evaluated the enzyme sensitivity to Pyr when both the major activator and inhibitor were present. For that purpose, the enzyme was assayed when it was partially inhibited with 50 µM AMP, and in presence of 100 µM Fru-1,6-P_2_. Under this condition, which maximizes the sensitivity towards those effectors, Pyr activated the enzyme 4-fold, with saturation kinetics slightly deviated from a hyperbolic behavior (*n*_H_ 1.2) (Figure 5). Here, the *A*_0.5_ was 2.76 mM, indicating that in presence of AMP and Fru-1,6-P_2_ the apparent affinity for Pyr was one order of magnitude higher in absence of other effectors. Remarkably, Pyr has a very significant effect on the partially inhibited enzyme. Noteworthy, the affinity for Pyr in these conditions drops to levels that are lower than the reported intracellular concentration, highlighting its putative physiological role [21].

Taken together, results presented *ut supra* show that Pyr has the merit to be considered an indirect or ancillary, yet important activator of *E. coli* ADP-Glc PPase. The keto acid is remarkably involved in augmenting the sensitivity of the enzyme to its main activator (Fru-1,6-P_2_) and decreasing the inhibition by AMP. Consequently, it further increases *V*_max_ and the affinity for substrates. Reciprocally, the affinity of the enzyme toward Pyr is greatly increased at very low concentrations of Fru-1,6-P_2_. A scenario can be proposed where Pyr behaves as a chief modulator of the allosteric regulation of the enzyme, mainly exerting its action by finely orchestrating the effect of the main allosteric effectors.

## Discussion

ADP-Glc PPases from diverse sources are classified according to their allosteric effectors [1], [2]. The *E. coli* enzyme is mainly activated by Fru-1,6-P_2_ and inhibited by AMP. Pyr has been identified as a major allosteric activator of other ADP-Glc PPases where AMP, ADP and/or P_i_ are inhibitors [1], [2]. There is an important amount of work that has been done regarding the characterization of Pyr as effector, mainly with the *A. tumefaciens* enzyme. Despite the fact that the crystal structure of the *A. tumefaciens* enzyme is available [7], it is not known where the Pyr binding site is. Therefore, it is not possible to do proper structural comparisons with the activator bound. This is an area that deserves further exploration. In other ADP-Glc PPases, Pyr shares its activation effect with six-carbon molecules such as Fru-6P [1], [2]. In this work, we found that Pyr has an important effect on the *E. coli* enzyme, which is in a class that traditionally has not been considered to have Pyr as an effector. Important elements to evaluate the significance of the Pyr effect are the 5-fold increase in *V*_max_ and the enhancement of apparent substrate affinities, mainly ATP. The *A*_0.5_ for Pyr was 25 mM; however, the most relevant effect is based on its interaction with the other regulators.

As demonstrated with different mutant strains of *E. coli* and *S. typhimurium*, there is a clear correlation between the apparent affinity of the enzyme for sub-millimolar concentrations of Fru-1,6-P_2_ and the ability to accumulate glycogen when compared to the wild type strain [30]. In this work, sub-saturating concentrations of Fru-1,6-P_2_ significantly increased the apparent affinity for Pyr, which highlights the existence of a cross-talk between these two effectors. This synergy would enhance the physiological effect. For instance, at 50 µM Fru-1,6-P_2_, Pyr *A*_0.5_ decreased 5-fold to reach a 5 mM value, which is within physiological range [21]. The reciprocal situation was also observed where 2.5 mM Pyr reduced in half the *A*_0.5_ for Fru-1,6-P_2_. Moreover, the apparent substrates affinities also increased by a synergistic effect between Fru-1,6-P_2_ and Pyr. We are unable to compare this phenomenon in the *E. coli* enzyme with other ADP-Glc PPases since this is the first time the effect of two synergistic allosteric activators in this family is reported. Nevertheless, our findings suggest that the regulatory fine-tuning of other ADP-Glc PPases should be revisited. Studies combining two activators, even some that may have been considered weak or non-physiological, will be helpful to verify whether this synergistic behavior is common to the enzyme from other diverse species.

We postulate that the interplay between Pyr and Fru-1,6-P_2_ has key implications at the physiological level. The “weak effect”, *per se*, that Pyr has on the enzyme becomes much more relevant in the presence of Fru-1,6-P_2_. Intracellular Pyr concentration was reported to be 7–14 mM [21], which is in fact in the range of the *A*_0.5_ for this metabolite in presence of very low Fru-1,6-P_2_ concentrations.

Another important feature is that Pyr diminished AMP inhibition. Given that this inhibition occurs only in presence of Fru-1,6-P_2_ [5], and considering that the latter interacts synergistically with Pyr, most likely the Pyr effect is indirect. That is, Pyr enhances the Fru-1,6-P_2_ activation, making the *E. coli* ADP-Glc PPase less sensitive to the inhibitor. In addition, Pyr completely reverses the (partial) AMP inhibition with an *A*_0.5_ of 2.76 mM (the smallest value observed in this work for Pyr).

From a mechanistic point of view, it is important to highlight that the synergistic effect between Pyr and Fru-1,6-P_2_ implies that they do not compete. Hence, they must be binding to two non-overlapping, but interacting allosteric sites. A traditional view of the promiscuity of this enzyme was that the allosteric site could accommodate different negatively charged molecules [1]. However, our results indicate that Pyr is not a promiscuous ligand that binds to the Fru-1,6-P_2_ allosteric site, but that it binds to a distinct site in the enzyme. This opens a new perspective to understand the evolvability of the ADP-Glc PPase family.

**Figure 6:**
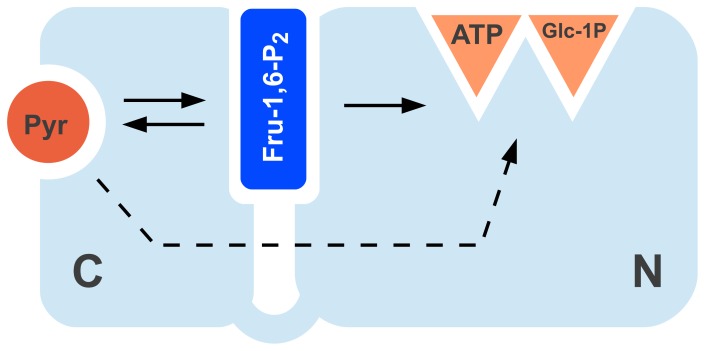
Proposed dual allosteric model for *E. coli* ADP-Glc PPase. The two main domains of the enzyme are illustrated by C and N. Catalysis occurs in the N-domain where the active site for ATP and Glc-1P is located. Full and dashed arrows indicate a strong and weak positive interaction, respectively.

The presence of a putative Pyr site explains the results observed with chimeric constructs between *E. coli* and *A. tumefaciens* [5]. The C-terminus had a major role in determining the affinity for Pyr, whereas both N- and C-domains shared the effect on Fru-1,6-P_2_ [5]. In addition, it is known that in the N-domain Lys^39^ interacts with the activator Fru-1,6-P_2_ [31]. This agrees with previous works on other ADP-Glc PPases where important residues for the interaction with the hexose-phosphate activator were found in both the N- and C-domain [7], [32]. On the other hand, those residues were not critical for Pyr activation [7], [32]. If we accept previous evidence that both domains interact with Fru-1,6-P_2_ [11], [33] and Pyr mainly with the C-terminus [5], [34], we can postulate the activation scheme in Figure 6 for the *E. coli* ADP-Glc PPase. In that scheme, Pyr binds to the C-domain and as a consequence, it activates the enzyme by a direct interaction with the catalytic site present in the N-domain. In addition, Pyr allosterically enhances the Fru-1,6-P_2_ binding to a site located in the interface between both domains. Finally, Fru-1,6-P_2_ transmits the allosteric signal to the active site. The increase of Fru-1,6-P_2_ apparent affinity implies an indirect regulatory effect from Pyr.

If we consider Pyr as a “new” effector, the *E. coli* ADP-Glc PPase regulatory scenario will become more similar to the enzyme from *A. tumefaciens,* which is activated by Fru-6P and Pyr [26] and from anoxygenic bacteria (e.g. *Rhodobacter* spp.) that are regulated by Pyr and an hexose-P (Fru-6P and/or Fru-1,6-P_2_) [1]. In those examples, the main difference from *E. coli* ADP-Glc PPase is that kinetic parameters for Pyr and the other effector are more similar [1], [35], [36]. It is very possible that a Pyr site may be present in many different types of bacteria rather than having a promiscuous binding to the hexose-phosphate site. The sensitivity for Pyr may have been “tuned” up or down by evolution according to the metabolic scenario.

As a whole, our results support the hypothesis that the *E. coli* ADP-Glc PPase is concurrently activated by both Pyr and Fru-1,6-P_2_ and also regulated by AMP levels. This multi-regulated mechanism reflects how the enzyme should operate in the actual metabolic environment in *E. coli* [11], [17]. There are two important aspects of Pyr regulation: (i) the enzyme in presence of activator and inhibitor (Fru-1,6-P_2_ and AMP, respectively) has a higher affinity for the keto acid; and (ii) even at sub-saturating concentrations, Pyr enhances the sensitivity of the enzyme for Fru-1,6-P_2_ activation. It could be proposed that Pyr orchestrates the activation of the main effector Fru-1,6-P_2_, working as an “activator of an activator” and playing consequently as a fine tuning modulator. Results obtained in this work will also help to understand the interplay between activators for other ADP-Glc PPases where more than one effector was reported. This poses an interesting case of allosterism where one metabolite facilitates the action of another allosteric activator and consequently organizes a global fine-tuned modulatory network.
